# Association Between Serum Vitamin D Levels and Body Mass Index Status: A Cross-Sectional Study at King Khalid Hospital, Jeddah, Saudi Arabia, From 2019 to 2020

**DOI:** 10.7759/cureus.46927

**Published:** 2023-10-12

**Authors:** Abdulaziz F Alharbi, Mohsin Ali, Yazeid I Alrefaey, Hossein A Filimban, Abdulrhman S Alhwaity, Ahmad Alamoudi, Muhammad A Khan

**Affiliations:** 1 Medical School, King Saud Bin Abdulaziz University for Health Sciences College of Medicine, Jeddah, SAU; 2 Family Medicine, Royal College of General Practitioners, London, GBR; 3 Family Medicine, Royal College of Physicians, London, GBR; 4 Family Medicine, King Saud Bin Abdulaziz University for Health Sciences College of Medicine, Jeddah, SAU; 5 Urology, King Saud Bin Abdulaziz University for Health Sciences College of Medicine, Jeddah, SAU; 6 Pediatrics, King Abdulaziz Medical City in Jeddah, Jeddah, SAU

**Keywords:** low vitamin d, obese patient, male sex, body mass index: bmi, serum vitamin d levels

## Abstract

Introduction

Low vitamin D levels have been associated more with overweight and obese people in previous studies. One of the possible explanations behind this association is the lipophilic property of vitamin D that causes the vitamin to deposit in adipose tissue and reduces the serum concentration of the vitamin, which still warrants further evaluation.

Objective

This study estimated the association between serum vitamin D levels and body mass index (BMI) among male patients in Jeddah, Saudi Arabia.

Methods

This is a cross-sectional study of male patients that was carried out in King Khalid Hospital, Jeddah, Saudi Arabia. Patients were included between October 2019 and November 2020.

Results

We concluded that almost half of the patients, 1,132 (48.3%), had adequate vitamin D, followed by 773 (33%) with vitamin D inadequacy, and only 288 (12.3%) had vitamin D deficiency. There was a significant relationship between age and vitamin D levels; younger people had lower vitamin D levels compared to older people (p<0.001). It was found that there was no significant relationship between BMI categories and vitamin D levels (p>0.05).

Conclusion

According to the findings of the current study, there was no discernible relationship between serum vitamin D levels and BMI. However, there was a substantial correlation between age and vitamin D levels, with younger age groups having lower amounts than older individuals. Future studies should adopt a prospective design encompassing multiple centers and preferably include the risk factors for developing vitamin D deficiency, such as sun exposure, dietary habits, comorbidities, etc. Such studies can provide a more accurate assessment of the association between BMI and vitamin D levels.

## Introduction

Body mass index (BMI) is an equation to identify the varying status of the body, including underweight, normal weight, overweight, and obesity. Diseases may reflect differently on the status of BMI. For example, one of the global health problems that has a major impact on morbidity and mortality is obesity [1]. Moreover, many countries have witnessed a rapid increase in the prevalence of obesity, making it a global issue [1]. Being overweight (BMI: 25-29.9 kg/m^2^) and obese (BMI: 30 kg/m^2^) are risk factors for many somatic disorders such as cardiovascular diseases, type 2 diabetes mellitus, and different types of cancer, which may cause serious health concerns [2-4]. Furthermore, low serum vitamin D levels have been recently recognized to be associated with obesity and an overweight BMI. Research by Lee et al. concluded that overweight and obese patients with low vitamin D levels may require higher doses of vitamin D to compensate for the deficiency [5]. In the past few years, the impact of vitamin D levels on different conditions has been investigated. A review by Wang S and Wang H points out the importance of a favorable level of vitamin D in the normal functioning of the cardiovascular system [6-7]. Moreover, a lot of conditions may affect the level of vitamin D, and excess body weight can be a valid reason [8-10]. The fat in the body may work as a storage for the lipophilic vitamins, therefore reducing their bioavailability due to their deposition in the adipose tissues [11]. Another well-known aspect of low vitamin D levels is lower than average skin exposure to the sun because ultraviolet radiation is one of the main sources of vitamin D [12].

In Saudi Arabia, females usually cover their skin when they go outside; thus, it will be difficult to determine whether obesity is the main reason for their low vitamin D levels. To avoid this conflict, this study only focuses on male patients. The relationship between vitamin D level and BMI is the focus of this study, and due to the lack of similar studies in the western region of Saudi Arabia, our study aimed to identify the association between various BMI statuses and serum vitamin D levels. In primary care and hospital settings, there have been a lot of requests recently to check vitamin D levels. Some of these requests are appropriate, and some are not. This study will help assess such requests and will help to see if the BMI could be used to decide whether a request for checking vitamin D levels could have any implications. Therefore, we were assessing based on the BMI of the patient present in a community or hospital setting, which could give a clue as to whether vitamin D testing would be appropriate or not and what the cost would be.

## Materials and methods

Study design

This is a cross-sectional study of male patients that was conducted at King Khalid Hospital, Jeddah, Saudi Arabia. Patients who had done vitamin D level testing and BMI recording between the periods of October 2019 and November 2020 were included.

Study population

This study included a total of 2,345 male patients. These patients met the inclusion criteria, which were: adult male patients (aged 18 years or older), not in any form of vitamin D supplementation, serum vitamin D level measured, and a BMI recorded (except for 219 patients who did not have their BMI recorded and were excluded from the comparison). Patients with diagnosed kidney disease were excluded. Patients were categorized into four different categories based on their vitamin D level according to reference ranges from the National Academy of Medicine in the US (<30 nmol/L: deficiency, 30-49 nmol/L: inadequate, >49 nmol/L: adequate, >125 nmol/L: potential adverse effects). By using the US BMI reference number, BMI was classified as (<18.5 kg/m^2^: underweight, 18.5-24.9 kg/m^2^: normal range, 25-29.9 kg/m^2^: overweight, 30-34.9 kg/m^2^: obesity class I, 35-39.9 kg/m^2^: obesity class II, >39.9 kg/m^2^: obesity class III).

Data collection

The data were collected with the help of the Research Data Management (RDM) team at King Abdullah International Medical Research Center (KAIMRC). The RDM has an established database of coded variables (lab tests, diagnoses, medications, etc.). They extracted the pre-coded data from the patient’s medical records electronically. No interview or contact was conducted with the patients. Study variables were age, sex, serum vitamin D level, BMI, and date of recording the vitamin D level and BMI.

Ethical considerations

Confidentiality was maintained throughout the study, as no identifier, name, or medical record number was collected. This study was approved by the Institutional Review Board (IRB) of KAIMRC (IRB approval number: IRB/1396/22).

Statistical analysis

In this study, categorical variables were presented as frequency and percentages, while numerical variables were presented as mean and standard deviation. We used the ANOVA test when comparing numerical and categorical variables, along with a 95% confidence interval. Analysis was performed using the IBM SPSS software version 20.0 (IBM Corp., Armonk, NY); statistical significance was set at p<0.05.

## Results

A total of 2,345 male patients were included in the study. The mean age of the patients was 51.1 years (SD=17.2). The mean vitamin D level among them was 62.9 nmol/L (SD=36.9) and their mean BMI was 28.8 kg/m^2^ (SD=6.1). According to the BMI categorization, the number of overweight patients was the highest with 788 (37.1%) patients, followed by obesity class I with 507 (23.8%) patients, while underweight patients were the lowest at 48 (2.3%). When checked for the vitamin D category, almost half of the patients, i.e., 1,132 (48.3%), had adequate vitamin D, followed by 773 (33%) with vitamin D inadequacy, and only 288 (12.3%) had vitamin D deficiency (Table 1).

**Table 1:** Baseline characteristics of the patients (N=2345) BMI: body mass index; SD: standard deviation; CI: confidence interval *BMI: total number () Significance was set at p<0.05.

	N	Mean	SD
Age	2345	51.1	17.23
Vitamin D	2345	62.9	36.95
BMI	2126	28.8	6.13
		n	%
*BMI (n=2126) (kg/m^2^)			
	Underweight	48	2.3
	Normal	498	23.4
	Overweight	788	37.1
	Obesity class I	507	23.8
	Obesity class II	184	8.7
	Obesity class III	101	4.8
Vitamin D (n=2345) (nmol/L)			
	Vitamin D deficiency	288	12.3
	Vitamin D inadequate	773	33.0
	Vitamin D adequate	1132	48.3
	Vitamin D: potential adverse effects	152	6.5

There was a significant difference between BMI classes and their mean age; normal, overweight, obesity class I, and obesity class II had a higher mean age than underweight and obesity class III (p<0.001). Vitamin D deficiency had a significantly lower mean age as compared to vitamin D adequacy, vitamin D inadequacy, and vitamin D potential adverse effects (p<0.001). It was found that there is no association between vitamin D level and BMI categories (p>0.05) (Tables 2-3).

**Table 2:** Comparisons between BMI categories using the ANOVA test BMI: body mass index; SD: standard deviation; CI: confidence interval Significance was set at p<0.05.

BMI	N	Mean Vitamin D (nmol/L)	SD	95% CI		p
Underweight	48	59.5	45.5	46.3	72.7	0.536
Normal	498	63.0	36.5	59.7	66.2	
Overweight	788	64.3	38.7	61.6	67.0	
Obesity class I	507	64.0	37.0	60.8	67.2	
Obesity class II	184	62.2	32.9	57.4	67.0	
Obesity class III	101	57.3	33.5	50.6	63.9	
BMI	N	Mean Age	SD	95% CI		p
Underweight	48	39.2	19.9	33.4	45.0	<0.001
Normal	498	49.6	19.4	47.9	51.4	
Overweight	788	51.7	16.3	50.6	52.9	
Obesity class I	507	53.7	15.9	52.3	55.1	
Obesity class II	184	53.2	14.8	51.0	55.3	
Obesity class III	101	47.8	15.7	44.7	50.9	

**Table 3:** Comparison between Vitamin D categories using the ANOVA test BMI: body mass index; SD: standard deviation; CI: confidence interval Significance was set at p<0.05.

Vitamin D level	N	Mean BMI (kg/m^2^)	SD	95% CI		p
Vitamin D deficiency	255	28.5	7.2	27.6	29.4	0.107
Vitamin D inadequate	702	29.0	6.4	28.6	29.5	
Vitamin D adequate	1028	28.9	5.7	28.5	29.2	
Vitamin D: potential adverse effects	141	27.7	5.6	26.8	28.7	
Vitamin D level	N	Mean Age	SD	95% CI		p
Vitamin D deficiency	288	46.0	18.2	43.9	48.1	<0.001
Vitamin D inadequate	773	49.9	17.1	48.7	51.2	
Vitamin D adequate	1132	52.9	16.9	52.0	53.9	
Vitamin D: potential adverse effects	152	52.5	16.6	49.8	55.1	

## Discussion

Serum 25-hydroxyvitamin D level is considered the best laboratory assessment for vitamin D status. Moreover, the trend of vitamin D level testing has been increasing as of late. Yet, the definition of vitamin D deficiency is still debated [13]. The Endocrine Society in Washington uses the cutoff level of <20 ng/mL (50 nmol/L) for vitamin D deficiency, while the National Academy of Medicine cutoff level for vitamin D deficiency is <12 ng/mL (30 nmol/L) [14,15]. Globally, vitamin D deficiency affects approximately 1 billion people [16]. The impact of this condition varies between regions, with the Middle East region being influenced the most [16]. In the United States, the estimated prevalence of vitamin D deficiency is 35% in the adult population, while in countries such as India, Pakistan, and Bangladesh, the estimated prevalence of vitamin D deficiency is over 80% of the adult population [16,17]. This difference could be related to extensive skin coverage and a higher content of melanin in these populations [16,17]. In our study, we used 30 nmol/L as a cutoff number for vitamin D deficiency and found that only 12.3% had vitamin D deficiency. In a meta-analysis done in Saudi Arabia, which included 16 studies from five different regions, the percentage of vitamin D deficiency was found to be approximately 60% [18]. This contrast could be attributed to multiple factors, one of which is the selected population in our study. Female patients were excluded from our study, while in the meta-analysis, they comprised about 62% of the population. Another contributing factor is the number of subjects included in the study, for which our study had 2,345 patients compared to the 20,787 patients found in the meta-analysis. A small proportion of our patients, around 6.5%, had elevated vitamin D levels that exceeded 125 nmol/L, and according to the National Academy of Medicine, these levels could potentially lead to adverse effects [15]. Taylor et al. published a paper regarding vitamin D toxicity due to inappropriate practices and found that the possible causes were formulation errors, inappropriate prescribing or dispensing, and improper administration of vitamin D [19]. The population that has an elevated risk of developing this condition is those of extreme ages, and the incidence is likely to increase due to growing interest in vitamin D testing and supplement prescription.

The status of BMI, particularly obesity, was also identified as a risk factor for having low serum vitamin D [20]. In our study, the mean BMI of vitamin D deficiency patients was 28.4 kg/m^2^; however, we were not able to find an association between vitamin D categories and BMI categories. Pereira-Santos et al. found that vitamin D deficiency was associated with obesity regardless of age and the cut-off level of vitamin D deficiency [20]. In their meta-analysis, which included 22 studies, the prevalence of vitamin D deficiency was 35% higher in obese subjects compared to the eutrophic group and 24% higher than in the overweight group [20]. Moreover, a study in Saudi Arabia found that there was an association between high BMI, low vitamin D levels, and diabetes. They found that only 26% of people with a normal BMI had vitamin D deficiency, while vitamin D deficiency was noticed in 90% of obese patients [21]. Furthermore, they also found that elevated levels of cholesterol and fasting blood glucose increase the risk of vitamin D deficiency [21].

Low vitamin D levels might predispose the individual to multiple health problems [16,20]. These individuals are more likely to develop bone diseases, fragility fractures, and chronic diseases such as asthma [16,22]. Although there are some controversies regarding the association between vitamin D and cancer, most of the evidence suggests that low vitamin D levels are associated with some cancer types, such as breast cancer, ovarian cancer, and glioblastoma [23].

Several theories were developed linking a person’s BMI to their vitamin D level. The first theory is the sequestration theory, in which vitamin D, a fat-soluble hormone, gets deposited in the adipose tissues, which leads to low circulating levels [24]. Volumetric dilution is considered one of the most probable mechanisms for lower serum vitamin D concentrations in obese patients. These people tend to have a higher volume of fat, muscle, and liver compartments, which vitamin D gets distributed into, leading to a low serum concentration of vitamin D [25].

One of the limitations of this study is that it is a single-center experience conducted at a tertiary hospital. Including other healthcare centers with a variety of healthcare settings could change the outcome. Moreover, risk factors for developing vitamin D deficiency, such as sun exposure, dietary habits, and comorbidities, were not included in the study. Future studies with a prospective design encompassing multiple centers and possibly including the risk factors for low vitamin D levels can provide a more accurate assessment of the association between vitamin D levels and BMI.

## Conclusions

The main purpose of the study was to identify any association between vitamin D levels and BMI. While no such association was identified, an observation was noted while analyzing the data that vitamin D levels varied according to the age groups, as the younger age group had lower vitamin D levels compared to older people who did not use supplements. This was an unexpected finding. For a better estimation of the association between vitamin D level and BMI, future studies should include risk factors for developing vitamin D deficiency, such as sun exposure, dietary habits, comorbidities, etc. Although this study has a sufficient sample size of 2,345 participants, a prospective study design will help avoid any recall or collection bias.
